# Electronic Diagnostic Support in Emergency Physician Triage: Qualitative Study With Thematic Analysis of Interviews

**DOI:** 10.2196/39234

**Published:** 2022-09-30

**Authors:** Matthew Sibbald, Bashayer Abdulla, Amy Keuhl, Geoffrey Norman, Sandra Monteiro, Jonathan Sherbino

**Affiliations:** 1 McMaster Education Research, Innovation & Theory (MERIT) Program Department of Medicine McMaster University Hamilton, ON Canada; 2 McMaster Education Research, Innovation & Theory (MERIT) Program Department of Health Research Methods, Evidence & Impact McMaster University Hamilton, ON Canada

**Keywords:** electronic differential diagnostic support, clinical reasoning, natural language processing, triage, diagnostic error, human factors, diagnosis, diagnostic, emergency, artificial intelligence, adoption, attitude, support system, automation

## Abstract

**Background:**

Not thinking of a diagnosis is a leading cause of diagnostic error in the emergency department, resulting in delayed treatment, morbidity, and excess mortality. Electronic differential diagnostic support (EDS) results in small but significant reductions in diagnostic error. However, the uptake of EDS by clinicians is limited.

**Objective:**

We sought to understand physician perceptions and barriers to the uptake of EDS within the emergency department triage process.

**Methods:**

We conducted a qualitative study using a research associate to rapidly prototype an embedded EDS into the emergency department triage process. Physicians involved in the triage assessment of a busy emergency department were provided the output of an EDS based on the triage complaint by an embedded researcher to simulate an automated system that would draw from the electronic medical record. Physicians were interviewed immediately after their experience. Verbatim transcripts were analyzed by a team using open and axial coding, informed by direct content analysis.

**Results:**

In all, 4 themes emerged from 14 interviews: (1) the quality of the EDS was inferred from the scope and prioritization of the diagnoses present in the EDS differential; (2) the trust of the EDS was linked to varied beliefs around the diagnostic process and potential for bias; (3) clinicians foresaw more benefit to EDS use for colleagues and trainees rather than themselves; and (4) clinicians felt strongly that EDS output should not be included in the patient record.

**Conclusions:**

The adoption of an EDS into an emergency department triage process will require a system that provides diagnostic suggestions appropriate for the scope and context of the emergency department triage process, transparency of system design, and affordances for clinician beliefs about the diagnostic process and addresses clinician concern around including EDS output in the patient record.

## Introduction

Diagnostic error is common in emergency departments [1-4], prolonging encounter times [5] and the length of stay [3] and increasing morbidity and mortality [2,3,5]. When systematically studied, cognitive factors (ie, how clinicians think) are frequently cited as an underlying cause [6]. When a diagnosis is missed, simply “not thinking of it” tops the list for causes [7]. The use of electronic differential diagnostic support (EDS) has emerged as a solution. EDS systems are decision aids that suggest a differential diagnosis (ie, a list of potential diagnoses) based on inputted data, allowing clinicians to be primed to potential diagnoses, thereby reducing the chance that they “do not think of it” [8-11]. Multiple studies have shown small but significant increases in diagnostic accuracy, using a variety of different EDS systems, with clinicians of different experience levels [8,11-14]. EDS increases the number of diagnostic hypotheses and the probability of the correct diagnosis being in the differential [8,11,13-15]. These benefits were present regardless of whether the EDS was used before or after the clinician had a chance to examine all of the available information [11].

In the emergency department, embedding EDS within a physician triage process holds promise to maximize the benefits of EDS. First, EDS flags relevant life-threatening diagnoses to clinicians, allowing a “must-not-miss list” to be at the top of their mind. Although clinicians are often trained to think about worst case scenarios, these life-threatening diagnoses are still occasionally missed or delayed. Prompting by EDS around multiple life-threatening diagnoses could facilitate timely intervention where delays matter (eg, antibiotics for potential meningitis in a patient presenting with altered consciousness). Second, physician triage directs up-front investigation, where EDS prompting may improve the range of investigations [13] with the potential to decrease emergency department visit time and improve the specificity of discharge diagnosis.

However, clinician adoption of EDS has been limited [10]. Prior work exploring attitudes about the feasibility and acceptability of EDS has identified that the additional time required to use an EDS was a deterrent [16]. Automating EDS data entry is a promising but untested approach, especially if the system can access data streams from the electronic medical record [17,18]. Such automation is possible within emergency department triage processes, where information collected by a triage nurse can be fed into an EDS system capable of natural language processing, providing subsequent clinicians with an EDS-supplied differential diagnosis.

However, it is unclear whether clinicians would be accepting of this type of approach, even if it were convenient. A human factors review highlights the critical influence of clinician perception and trust of tools such as EDS as being important in their adoption within health care contexts [17]. Clinician perception around the perceived quality of EDS suggestions could influence their willingness to use it. Similarly, trust in the EDS system and the relative control over data fed into the EDS system also shapes the acceptance of the technology. In fact, recent evidence raises concern that clinicians who are mistrusting of EDS systems do not appear to benefit from them [12]. To assess the acceptability of integrating EDS within the triage process, we conducted a qualitative study of emergency department triage physicians to identify their perceptions and concerns with this approach.

## Methods

We conducted a qualitative study, interviewing physicians involved in an EDS-aided emergency department triage process.

### Setting

This study was conducted at a tertiary care emergency department of an academic teaching hospital with a physician-supported triage process. In this setting, patients were assessed in a triage process that includes a triage nurse and a triage physician, before being assigned to a zone for a more thorough assessment. Patients were registered by a triage nurse who noted the chief complaint and vitals. Patients were subsequently assessed by a triage physician who performed and documented an abbreviated history and physical examination. The triage physician assigned patients a zone within the emergency department based on the severity of illness, established a working diagnosis or differential diagnosis, and ordered initial investigations and time critical interventions. Patients were subsequently moved to a different area of the emergency department based on the triage physician’s decision, to be assessed by the most responsible emergency department physician within the assigned zone. This process allowed initial investigations and management to start even if the physician assigned to the patient’s zone is busy. The triage physician process is not meant to be comprehensive; the full history and physical examination and all subsequent investigations, management, and disposition determinations are performed by the most responsible physician. Triage physicians work quickly to keep up with the emergency department volumes, typically assessing 12-15 patients per hour. Trainees are not involved in the triage process given the need to keep pace with the volume of patients presenting to the emergency department. All triage physicians are fully qualified, independently practicing emergency department physicians.

### EDS-Aided Emergency Department Triage Process

We developed a rapid human prototype, using a research associate, to simulate automated EDS integration into the triage process of a busy emergency department. This approach allowed triage physicians participating in the triage process to access EDS without having to input data themselves into the system. We chose the Isabel system (Isabel Healthcare) as it was one of the most frequently studied EDS platforms, with minimal time investment required for use [10,11,16]. A research associate (BA), who is a medical doctor with emergency department training, entered data from the triage nurse note into the EDS system using a tablet. The Isabel system accepts patient age, gender, travel history, and symptoms, which are entered into textboxes. The system uses natural language processing to provide lists of potential diagnoses, flagging the diagnoses that are life-threatening. The research associate provided the triage physician with the EDS output via the tablet before the physician assessed each patient complaint in person. This process mimicked automated access to the EDS output based on the triage record within the electronic medical record.

### Recruitment

All emergency department physicians were involved via a rotating schedule with the triage process. All were invited to participate by email. Written consent was obtained. Participants were provided with a simulated EDS integration into the electronic medical record for the last hour of their triage shift. Each participant took part in a semistructured interview at the end of their triage shift.

### Interviews

Semistructured interviews were conducted by a research associate (BA), which were then transcribed verbatim from recordings. The interview guide included questions around the perceived role for EDS integration into the triage process, potential and actual advantages and disadvantages, any impact the system had on patient management decisions, opinions on how to best integrate the system into the workflow, and whether it would be advantageous for trainees to use the system.

### Analysis

We performed a direct content analysis of the semistructured interviews of emergency department physicians who had access to the EDS during a triage shift. Analysis was anchored within a human factors paradigm, highlighting the role of perception, usability, workload, and trust in automated electronic approaches [19]. Using the principles of direct content analysis [20], the analytic team (MS, JS, SM, AK, and BA) read the transcripts and engaged in a process of iterative open coding, followed by group discussion to inform subsequent coding. Following open coding, we engaged in axial coding to establish linkages between the data, informed by the human factors paradigm. The team met regularly during the analytic process to revise the interview guide and discuss interim analyses and emerging themes. Theoretical saturation was determined through analytic consensus, where empiric evidence supported themes of sufficient depth to advance understanding while maintaining practical relevance.

### Rigor

The research team consisted of varied perspectives including clinicians (MS, BA, and JS), educational scientists (MS, SM, and GN), and an educator (AK). We adopted a realist stance, recognizing that participant and contextual factors would influence our data set. We enhanced rigor through a purposeful sampling of practicing clinicians at a single busy center. The interviews were completed by a physician with sufficient content knowledge to understand usability challenges but without knowledge of the emergency department workflow to avoid in-group assumptions around technology adoption and mitigate social desirability bias that might moderate opinions. The interviewer kept field notes and a reflective diary. Transcripts were transcribed verbatim to avoid losing word choices and tone and were anonymized prior to analysis to preserve confidentiality and avoid bias from the analytic team. Throughout the analysis, the analytic team declared biases and assumptions and actively sought contrasting opinions from others involved in the analytic process.

### Ethics Approval

This study was approved by the Hamilton Integrated Research Ethics Board (#13926). All participants provided written informed consent.

## Results

### Participant Demographics and Themes

We conducted 14 interviews from 13 emergency department physicians, as described in Table 1.

One participant used the EDS for 2 shifts and underwent 2 interviews. Of the 13 physicians, the years in practice varied: 5 (38%) were within the first 5 years of practice, 4 (31%) within 5-10 years, 1 (7%) within 11-20 years, and 3 (23%) with greater than 20 years. In all, 6 (46%) physicians were certified as family medicine specialists with subspecialization in emergency medicine, and 7 (54%) were certified as emergency medicine specialists. In total, 4 themes were identified in the analytic process, as described in Table 2.

**Table 1:** Participant demographics.

Demographic			Participant (N=13), n (%)
**Years of experience**			
	<5	5 (38)	
	5-10	4 (31)	
	11-20	1 (7)	
	>20	3 (23)	
**Gender**			
	Female	2 (15)	
	Male	11 (85)	
**Specialty**			
	Family medicine with emergency medicine training	6 (46)	
	Emergency medicine	7 (54)	

**Table 2:** Themes identified by clinicians around incorporating electronic differential diagnostic support (EDS) systems in the emergency department.

Theme	Relevant human factors construct^a^	Description	Number of clinicians who made statements that supported this theme (N=13), n (%)
1. The quality of the EDS was inferred from the scope and prioritization of the diagnoses.	Perception and usability	Participants linked the value of the EDS to the types of diagnosis being suggested. Diagnoses that did not seem appropriate or were outside of the physician’s range of practice were suggested, which prompted clinicians to doubt the value of the EDS.	9 (69)
2. Trusting EDS differential diagnoses was linked to varied beliefs around the diagnostic process and potential for bias.	Trust and usability	Participants were concerned about the unintended and untested benefits of EDS. Some worried that it might introduce bias, whereas others wanted to see more evidence of its benefit.	10 (77)
3. Who benefits? Not me.	Perception and trust	Participants acknowledged that EDS could add value but found it hard to imagine that they would make an error that the EDS could correct.	13 (100)
4. Information flow between EDS and the electronic medical record	Usability and workload.	Participants believed that EDS should be able to use information in the medical record to provide a differential diagnosis, but that the differential diagnosis output of the EDS should not be automatically incorporated into the medical record. Including the EDS output could prompt an over investigation of diagnostic suggestions even when they are not appropriate to the context.	8 (62)

^a^Adapted from Asan and Choudhury [19].

### Theme 1: The Quality of the EDS Was Inferred From the Scope and Prioritization of the Diagnoses

A lot of [the suggested diagnoses] seemed very extraneous, I’m not totally sure where they got them from. There were a couple where...I don’t see a world that that’s what’s going on with this patient.

Participant #6

Participants passed judgment on the quality of the EDS based on whether the diagnostic suggestions were relevant to their context and scope of practice in the emergency department.

This could be Goodpasture’s [disease]...But do I think that that patient actually had any of those things? Absolutely not...Should I do a bunch of blood work? I don’t think that’s really my role.

Participant #13

The suitability of diagnostic suggestions was even tied to the patient’s location in the emergency department.

It was telling me, intestinal ischemia in a 25-year-old with abdominal pain...we would be able to see that within the first couple of seconds by looking at them. And they sure as hell wouldn’t be seen at triage, I’m sure they’d be in the resuscitation bay.

Participant #10

Similarly, participants assumed that the ordering of diagnoses on the list was related to probability, as this is a common clinical convention. The system’s utility was questioned when the ordering of the diagnoses did not align with participants’ clinical impression: “either that the most likely diagnosis was not even on the list or it was really far down, and they had very unlikely diagnoses closer to the top” (Participant #8).

Of note, EDS was used early in the emergency department visit where there was only limited information available based on the triage nurse’s intake process. This process was not readily apparent to participants, occasionally leading to poor perception of the EDS quality, particularly when patients were unable to articulate their symptoms well, presented with misleading or vague complaints, or did not disclose relevant past medical history up-front.

### Theme 2: Trusting EDS Differential Diagnoses Was Linked to Varied Beliefs Around the Diagnostic Process and Potential for Bias

Participants were skeptical about EDS adoption within the triage process, voicing concern around trusting the accuracy of the outputted differential diagnosis. These concerns were both around how EDS would influence the diagnostic process, as well as how EDS generated a differential diagnosis.

#### Subtheme 1: Influence on the Diagnostic Process

Participants were concerned about the potential for EDS to bias them: “it would actually be more useful if it told me stuff after I’d seen the patient to jog my memory, rather than going in biasing me a little bit” (Participant #10). They had strong, but divided, beliefs about whether the EDS should be used early or late in the diagnostic process to mitigate bias. The use of EDS up-front concerned some participants that their judgment would be “clouded,” “anchored,” or “biased,” whereas others saw it as a mechanism to “combat confirmation bias,” “think outside the box,” and “avoid tunnel vision.” Interestingly, the need to reduce bias was given as a justification for both positions.

#### Subtheme 2: Transparency Around How the EDS Generated a Differential Diagnosis

Many participants wanted to understand more about how the system created a differential diagnosis before adopting it in their practice or advocating for it to be embedded within the triage system: “I did not really find it that helpful, to be honest with you...First of all...I have no idea how the software works” (Participant #8). There were calls from participants to make explicit the algorithms used as well as a desire for empiric evidence that the algorithms improved the diagnostic process and reduced diagnostic error.

### Theme 3: Who Benefits? Not Me

Most participants did not see a personal benefit but endorsed the use of the EDS for less experienced colleagues. A small number of participants acknowledged that experienced clinicians might derive benefit, either through episodic use in situations where clinicians faced diagnostic uncertainty or through consistent use to identify situations where a diagnosis was simply overlooked.

I think it would be a great thing for learners or less experienced docs...sometimes it’s helpful...for someone who has experience in that, some of the differential diagnoses are things that might not have popped into my mind.

Participant #1

Participants had strong divergent opinions around whether learners would benefit from the EDS, with some believing that learners need to practice generating diagnoses on their own to avoid “spoon feeding them” and others believing that it would “overwhelm” them, which is in contrast with those believing it to be a good learning tool to “develop diagnostic acumen” and a way of reducing “cognitive overload” for learners. Opinions also diverged on whether it made learners safer: “It can help them to make sure they’re keeping a broad differential instead of narrowing in on and prematurely closing” (Participant #8) versus “If they’re being supervised, then I don’t see a reason for it...from a patient safety point of view” (Participant #5).

### Theme 4: Information Flow Between EDS and the Electronic Medical Record

Participants did not react adversely to the EDS drawing data from the electronic medical record. In fact, extracting data from the electronic medical record to automate EDS output was desirable, as long as the click burden was low. Participants wanted some control over this process to understand the data that were being fed to the EDS and, in some cases, wanted the system to only use the data that they personally collected or vetted.

However, participants had substantial concerns around the output of EDS being a part of the medical record. Uniformly, clinicians opposed the EDS output being documented in the medical record, citing medicolegal implications.

I think you could run into problems if there’s an AI-generated differential and your clinical gestalt disregards a couple of the points because you don’t think they’re likely. And then if there’s a bad outcome...being held liable because the AI-generated differential told you to consider such and such.

Participant #8

## Discussion

### Principal Findings

In this study, we provided EDS to emergency department physicians doing triage assessment using a researcher to simulate an automated system. We found that emergency department physicians were hesitant about adopting this approach with 4 themes emerging. First, quality was inferred by the scope and prioritization of the EDS output, which was not customized to the emergency department triage setting. Second, trust in the EDS was linked to participant beliefs and assumptions of the diagnostic process and would benefit from transparency and evidence around EDS function and outcomes. Third, participants were disinclined to consider themselves to be in the group of clinicians that would benefit from EDS, with divergent opinions on whether the EDS should be a tool for learners. Finally, participants wanted the ability to draw from the medical record to feed the EDS but insisted that the EDS output be kept out of the patient record.

This study adds to the literature as interventions aimed at reducing diagnostic error are rarely assessed in the workplace [21,22]. A recent narrative review highlighted that most interventions are studied under experimental circumstances and called for more investigation of interventions within the workplace settings [21]. Within the emergency department setting, second opinions, decision aids, guided reflection, and focused education have all been proposed to reduce diagnostic error, although most of these interventions have been evaluated in experimental settings [21]. Some data is available regarding the perceived benefit of checklists in a clinical workplace setting [21,22]. One study conducted by Graber et al [22] used a rapid cycle design process to iterate the checklist content in primary care settings. Clinicians identified situations where the checklist had changed the working diagnosis, unlike in this study of EDS where clinicians only hypothesized a benefit. Interestingly, both interventions involved similar content (eg, the specific checklist for chest pain is highly similar to the output provided by the EDS of a middle-aged man with chest pain). However, clinician exposure to the checklists was over a 2-month period, far greater than the single shift used in this study.

The clinician uptake of EDS was low in a different 3-month pilot, with the authors calling for the customization of the platform to better suit the primary care environment [23]. In particular, the platform suggests a level of diagnostic precision that may not be realistic for initial illness presentations with differential diagnoses being constructed with different precisions over the course, context, and type of clinician assessing the patient [24]. These concerns raised in the primary care setting are equally applicable to the emergency department. Diagnostic labels such as “chest pain not yet diagnosed,” “chest pain query pulmonary embolism,” and “rule out acute coronary syndromes” are common in the triage process. Similarly, discharge with the “test of time” using guidelines around when to return to the emergency department is a frequent strategy to avoid over investigation while allowing a serious illness to declare itself [25]. These approaches suggest that clinicians accommodate diagnostic uncertainty, with an incremental approach to diagnostic labeling and subsequent testing, which does not neatly fit the EDS platform used.

For those involved in EDS design, this study highlights areas where the EDS platform could be modified to facilitate clinician adoption in the emergency department. Clinicians seemed to lose faith in the EDS system when diagnostic suggestions did not fit the context or scope of their practice. This finding leaves open the possibility of greater clinician trust of EDS systems if suggestions were customizable to their environment and scope of practice. Similarly, the ergonomics of use were frequently highlighted. Attempts to automate EDS within the triage process or embed it within the electronic medical record should prioritize the transparency of the data being entered and allow single-click access at different stages of the diagnostic process, as clinicians are unlikely to agree on a common strategy for use.

For those advocating for EDS use in practice, several considerations are warranted. First, clinicians’ underlying beliefs and assumptions about the diagnostic process influenced their willingness to use the EDS and perceptions around its benefits. In some cases, these beliefs seemed to conflict with evidence that experienced clinicians can benefit from EDS [10] and that benefit is present both with early and late use in the diagnostic process [11]. Second, clinicians have a need to understand how the EDS works and the evidence for its effectiveness in practice. Although evidence of EDS improvements in diagnostic accuracy exists in controlled settings [10], it is unclear whether it is justified to subject EDS to the same level of evidence required for diagnostic tests or therapeutic strategies, as evidence in either of these paradigms is limited [26]. At minimum, increasing transparency around how an EDS generates a differential may enhance clinician adoption.

### Limitations

We highlight 2 important limitations. First, this study took place in a single setting, with a limited sample size and a single approach to embedding EDS within the triage process. This process allowed the uniformity of experience but limited generalizability to other settings. Second, the opinions and perceptions of clinicians can deviate from their behavior in practice, thus limiting the inferences that can be drawn.

### Conclusions

Using a research associate to mimic the integration of EDS into the triage process proved feasible. However, clinicians remain skeptical about the value of the EDS output in the triage process. Those interested in facilitating EDS adoptions should consider (1) whether the diagnostic suggestions provided are appropriate for the scope and context of the emergency department triage process, (2) how much transparency of the EDS system’s inner working is required to earn the trust of clinicians, (3) addressing clinician beliefs about the diagnostic process, and (4) addressing clinician concerns around including EDS output in the patient record.

## Abbreviations

EDS: electronic differential diagnostic support
